# Duodenal microbiota and weight-loss following sleeve gastrectomy and Roux-en-Y gastric bypass – a pilot study

**DOI:** 10.1186/s12893-023-02076-6

**Published:** 2023-06-26

**Authors:** Tomasz Stefura, Jakub Rusinek, Maciej Zając, Barbara Zapała, Tomasz Gosiewski, Agnieszka Sroka-Oleksiak, Dominika Salamon, Michał Pędziwiatr, Piotr Major

**Affiliations:** 1 Department of Medical Education, Jagiellonian University Medical College, Krakow, Poland; 2 Students’ Scientific Group at 2nd Department of General Surgery, Jagiellonian University, Medical College, Krakow, Poland; 3 Department of Clinical Biochemistry, Jagiellonian University Medical College, Krakow, Poland; 4 Department of Microbiology, Jagiellonian University Medical College, Krakow, Poland; 5 2nd Department of General Surgery, Jagiellonian University Medical College, Kopernika 21 St, 31-501 Kraków, Poland

**Keywords:** Obesity, Bariatric surgery, Sleeve gastrectomy, Microbiota

## Abstract

**Background:**

Bariatric surgery is the most effective method of morbid obesity treatment. Microbiota has many functions in human body and many of them remain to be unknown. The aim of this study was to establish if the composition of duodenal microbiota influences success rate of bariatric surgery.

**Methods:**

It was a prospective cohort study. The data concerning demographics and comorbidities was collected perioperatively. The duodenal biopsies were collected prior to surgery with the gastroscope. Then DNA analysis was conducted. The data connected to the operation outcomes was gathered after 6 and 12 months after surgery.

**Results:**

Overall, 32 patients were included and divided into two groups (successful – group 1 and unsuccessful – group 0) based on percentage excess weight loss after 6 months were created. The Total Actual Abundance was higher in group 0. In group 0 there was a significantly higher amount of Roseburia and Arthrobacter (*p* = 0.024, *p* = 0.027, respectively). Genus LDA effect size analysis showed Prevotella, Megasphaera and Pseudorhodobacter in group 1 to be significant. Whereas abundance of Roseburia and Arthrobacter were significant in group 0.

**Conclusions:**

Duodenal microbiota composition may be a prognostic factor for the success of the bariatric surgery but further research on the larger group is needed.

## Background

Over the last years obesity rose to become one of the most important health problems in the western countries, with World Health Organisation (WHO) declaring that we are facing an epidemic [1]. This led to developments and improvements in prophylaxis and treatment techniques, with bariatric surgery becoming the best solution for individuals suffering from morbid obesity, which are usually unable to reduce weight by other, non-invasive methods [2]. The term bariatric surgery refers most often to the two most popular surgical procedures—sleeve gastrectomy (SG) and Roux-en-Y gastric bypass (RYGB).

Microbiota and the impact it has on various functions of the human body is becoming an increasingly interesting area of research, with studies suggesting its’ impact on the brain (so-called gut-brain axis), vascular system and overall metabolic health [3, 4]. The gut microbiota appears to also play a role in determining the outcome of a bariatric surgery, however, this association is still unclear and requires further research [5]. There have been previously published studies on the effect the bariatric procedures have on the microbiota, but little to no research has been conducted on the role the duodenal microbiota plays in achieving satisfying weight loss after surgery [6].

The following is a pilot study trying to establish whether certain composition of duodenal microbiota is associated with higher success rate of bariatric surgeries defined as achieving satisfying weight loss after undergoing the procedure.

## Methods

### Study design

This prospective cohort study has been conducted in one academic hospital between 2013 and 2016. We decided on the following inclusion criteria: informed consent for participation in the study, meeting the qualification standards for bariatric surgery, age between 18 and 65 years. In order to be qualified for a bariatric surgery patients had to: have the body mass index (BMI) of ≥35kg/m^2^with other obesity-related comorbidities, or BMI ≥40kg/m^2 ^without comorbidities. RYGB or SG was chosen depending on the comorbidities such as hiatal hernia and coexisting inflammatory lesions in oesophagus. RYGB was suggested for patients with coexisting inflammatory lesions, while SG for patients without those lesions. Final decision concerning surgery was always made by patient after transparent presentation of advantages of each procedure [7]. Those criteria were adapted from the recommendations of The Metabolic and Bariatric Surgery Section of The Association of Polish Surgeons [8]. As for the exclusion criteria they were as follows: reported antibiotic or probiotic intake in the 30 days preceding the collection of samples, history of infections of the gastrointestinal tract, hyper or hypoactivity of the thyroid, cancer, immunodeficiency, inflammatory bowel diseases. Study design and data collection has been conducted in accordance with the Strengthening the Reporting of Observational studies in Epidemiology (STROBE) guidelines [9]. In the perioperative period anthropometric and clinical data, such as age, sex, perioperative body mass, BMI, comorbidities (respiratory disorders, dyslipidemia, liver steatosis, type 2 diabetes, hypertension, joint disorders, varicose veins) and American Society of Anaesthesiologists (ASA) class were collected. For the microbiota assessment the authors collected duodenal biopsies prior to operation with the use of a gastroscope. Endoscopy of the upper gastrointestinal tract is a routine procedure conducted before every bariatric surgery in our hospital. It includes the assessment of oesophageal, gastric, and duodenal mucosa pathology. We also obtained gastric mucosa tissue biopsies and rapid urease tests for Helicobacter pylori infection (Campylobacter-like organism (CLO) test). Gastritis or oesophagitis was the indication for proton pump inhibitor (PPI) treatment. In case of positive result of CLO test, we conducted eradication accordingly to the guidelines of the Polish Society of Gastroenterology for the diagnosis and treatment of Helicobacter pylori infection [7].

**Fig. 1:**
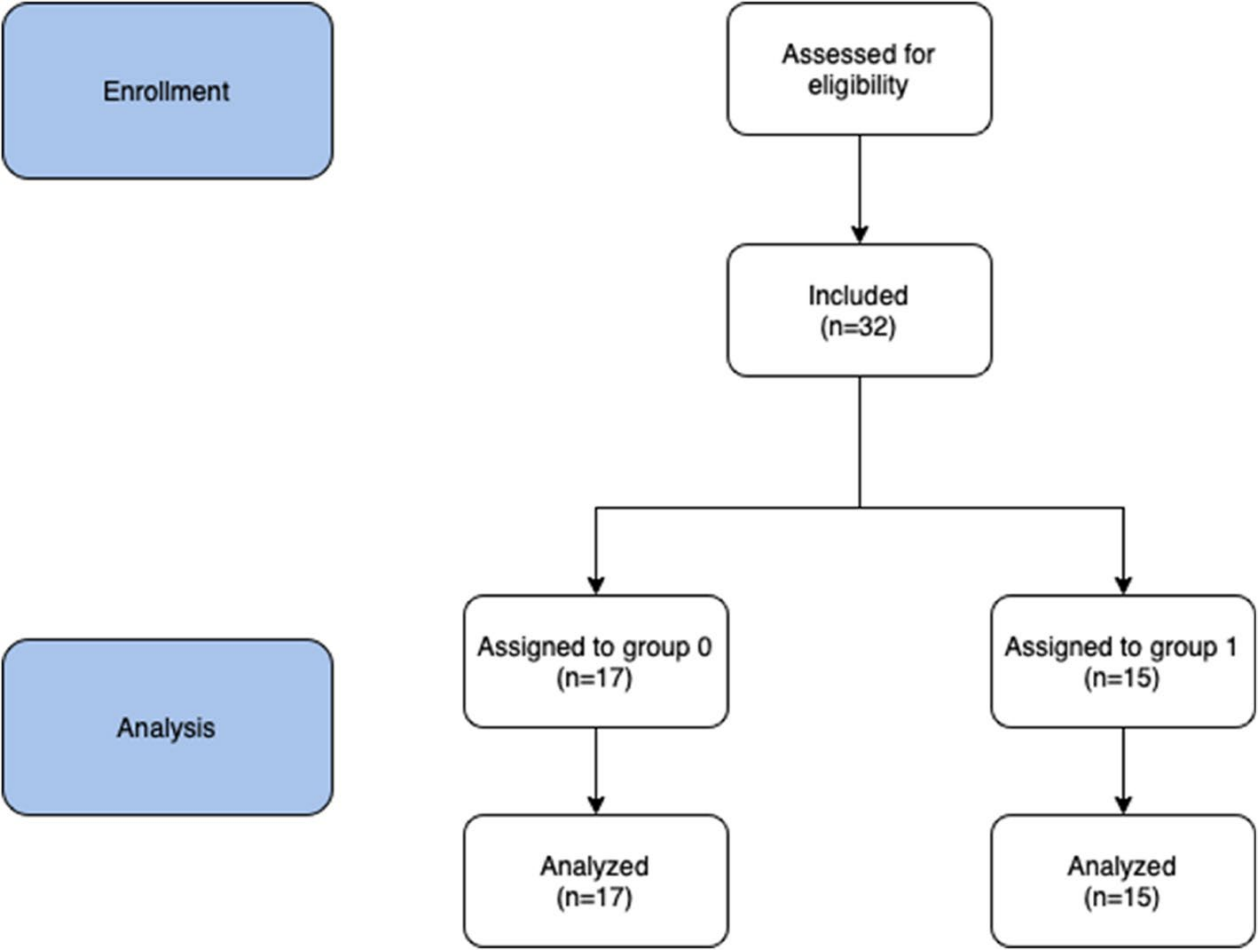
Flowchart of the study

Then DNA isolation, amplification and taxonomical analysis have been conducted. Postoperatively, the authors also recorded data concerning the course of the bariatric procedure—duration of the surgery, intraoperative and postoperative adverse events. Lastly, after 6 months and 12 months from the surgery, data connected with assessment of the outcomes was collected and computed – this included percentage excess BMI loss (%EBMIL), percentage excess weight loss (%EWL) and percentage total weight loss (%TWL). Based on the data after 6 months patients were assigned to two groups: group 1 (successful)—consisted of patients who achieved %EWL of 50% or more. The remaining patients were assigned to group 0 (%EWL < 50%). As it has been demonstrated in other studies, the criterion of achieving 50% %EWL is a valid indicator of successful bariatric procedure [10]. Figure 1 presents the flowchart of the study.

### Collection of duodenal biopsies and DNA isolation

To analyse the composition of duodenal microbiota we obtained duodenal mucosa tissue samples during routine preoperative gastroscopy for patients undergoing bariatric surgery. The descending part of the duodenum was sampled. Biological material obtained has been deep frozen and delivered to the laboratory where 32 duodenal biopsy samples were isolated and 5 sterile water samples were included as negative control. Bacterial DNA has been isolated using Mini Genomic Kit (A&A Biotechnology, Gdańsk, Poland) according to the procedure described by Gosiewski et al. [11]. Assessment of purity and concentration of the isolated DNA has been conducted with the use of NanoDrop (Thermo Scientific). The duodenum, especially the descending part is characterized by relatively low population of bacteria (compared to other sections of the gastrointestinal tract), which necessitated the use of nested-PCR method for increasing the specificity and sensitivity of isolated DNA. Specific primers for 16S rRNA gene with V3 and V4 regions have been described in Table 1 as well as the composition of reactive mixture and amplification programme.

**Table 1:** Used primers, amplification programme and reaction mixtures

Oligonucleotide Sequence (5’- > 3’)	Reaction mixture		Amplification Program	
F: ACGGCCNNRACTCCTAC R: TTACGGNNTGGACTACHV	Water	2.6 is *µl*	95 ºC	5 min
	Kappa	5.0 µl	95 ºC	15 s (× 40)
	Primer F (10 *µM*)	0.2 µl	48 ºC	20 s (40x)
	Primer R (10 *µM*)	0.2 µl	72 ºC	30 s (40 s)
	DNA	2.0 µl	72 $^\circ C$	5 min
F: CCTACGGGNGGCWGCAG R: GACTACHVGGGTATCTAATCC	Water	10.5 µl	95 ºC	5 min
	Kappa	12.5 µl	95 ºC	30 s (25x)
	Primer F (10 *µM*)	0.5 µl	55 ºC	30 s (25x)
	Primer R (10 *µM*)	0.5 µl	72 ºC	30 s (25x)
	DNA	1.0 µl	72 ºC	5 min

Products obtained from the amplification procedure were separated using electrophoresis to confirm the size of amplicon. All further proceedings were conducted in accordance with the MiSeq sequencer protocol (Illumina). Data gathered in the following study is available in the public domain and can be accessed with the use of the following link: https://portalwiedzy.cm-uj.krakow.pl/info/researchdata/UJCM2487d902aba44a418407170a85fdfb37/Record%2Bdetails%2B%25E2%2580%2593%2BResearch%2Bdata%2B%25E2%2580%2593%2BJagiellonian%2BUniversity%2BMedical%2BCollege?r=researchdata&ps=20&tab=&lang=en.

### Statistical analysis

The statistical analysis was done with the use of IBM SPSS Statistics version 28. Categorical variables are presented as number (n) and percentage (%). Chi-squared test was used to assess the differences between the groups considering categorical variables. When it comes to quantitative variables, their presentation is based on distribution. The Shapiro–Wilk test was run to explore the distribution. Normally distributed data is presented as mean and standard deviation (SD) whereas non-normally distributed data is presented as median with first and third quartile (Q1-Q3). Furthermore, the differences between groups were analysed with Student’s *t*-test (for normal distribution) and Mann–Whitney *U* test (for nonparametric data). Alpha diversity was analysed for two taxa – species and phylum with the usage of Chao1, ACE, Shannon and Simpson indices. Beta diversity was assessed for the same two taxa—Brey-Curtis index was used. Median total actual abundance and subtaxonomic abundance were calculated and presented as graphs. Linear discriminant analysis (LDA) which allows to distinguish which taxa best explains the differences between samples by conventional statistical tests, tests of biological consistency and effect relevance [12]. The groups were further divided based on the operation type (RYGB or SG) and the same microbiological analysis were conducted for those groups. LDA score > 2.0 was considered significant. The *p*-value < 0.05 was considered statistically significant.

## Results

### Demographic characteristics

The group of 32 patients was included into the study. Patients were allocated into two groups – group 0 (*n* = 17 (53.1%)) and group 1 (*n* = 15 (46.9%)). Overall, 37.5% of the study population was male. There was a statistically significant difference between two groups considering mean age – 38.47 (± 10.37) in group 1 vs. 45.82 (± 9.84) years in group 0 (*p* = 0.048). Other demographic parameters showed no significant difference between the groups. Mean maximal BMI in the study population was 48.97 (± 6.50) kg/m2 and mean BMI before surgery was 47.75 (± 6.60) kg/m2. Most frequent comorbidities were liver steatosis (occurred in 26 (81.3%) participants), dyslipidaemia (in 23 (71.9%) participants) and hypertension (in 23 (71.9%) participants). More data on demographic characteristics can be found in Table 2.

**Table 2:** Patients baseline characteristics

Characteristic	All (*n* = 32)	Group 0 (*n* = 17)	Group 1 (*n* = 15)	*p*-value
Male, n (%)	12 (37.5%)	6 (35.3%)	6 (40.0%)	1.000
Age (years), mean (SD)	42.38 (10.61)	45.82 (9.84)	38.47 (10.37)	**0.048**
Max. Weight (kg), mean (SD)	144.02 (27.21)	147.12 (24.72)	140.50 (30.27)	0.501
Max. BMI (kg/m2), mean (SD)	48.97 (6.50)	50.00 (6.00)	47.80 (7.05)	0.347
Weight loss before the surgery (kg), median (Q1-Q3)	2.50 (0.00—5.50)	3.00 (0.00—4.50)	0.00 (0.00—6.00)	0.911
BMI before the surgery (kg/m2), mean (SD)	47.75 (6.60)	48.93 (6.00)	46.42 (7.20)	0.292
Comorbidities				
GERD, n (%)	4 (12.5%)	2 (11.8%)	2 (13.3%)	1.000
Diabetes, n (%)	10 (31.3%)	6 (35.3%)	4 (26.7%)	0.712
Insulin resistance, n (%)	2 (6.3%)	2 (11.8%)	0 (0.0%)	0.486
Dyslipidaemia, n (%)	23 (71.9%)	11 (64.7%)	12 (80.0%)	0.444
Liver steatosis, n (%)	26 (81.3%)	13 (76.5%)	13 (86.7%)	0.659
Hypertension, n (%)	23 (71.9%)	13 (76.5%)	10 (66.7%)	0.699
Another cardiologic diseases, n (%)	3 (9.4%)	2 (11.8%)	1 (6.7%)	1.000
Respiratory disorders, n (%)	6 (18.8%)	2 (11.8%)	4 (26.7%)	0.383
Joints disorders, n (%)	16 (50.0%)	10 (58.8%)	6 (40.0%)	0.479
Varices, n (%)	6 (18.8%)	3 (17.6%)	3 (20.0%)	1.000
Smoking, n (%)	3 (9.4%)	2 (11.8%)	1 (6.7)	1.000
ASA scale, median (Q1-Q3)	2 (2–3)	2 (2–3)	2 (2–3)	0.710
ASA 1, n (%)	3 (9.4%)	1 (5.9%)	2 (13.3%)	0.770
ASA 2, n (%)	20 (62.5%)	11 (64.7%)	9 (60.0%)	0.770
ASA 3, n (%)	9 (28.1%)	5 (29.4%)	4 (26.7%)	0.770

### Perioperative results

Overall, SG was performed in 18 (56.3%) patients and RYGB was performed in 14 (43.8%) patients. There were no statistically significant difference between the groups concerning the bariatric procedures and its complications. Mean (SD) duration of surgery was 117.66 (42.07) min and 4 (12.5%) patients suffered from complications. More detailed information can be found in Table 3.

**Table 3:** Perioperative results

Characteristic	All (*n* = 32)	Group 0 (*n* = 17)	Group 1 (*n* = 15)	*p*-value
LSG, n (%)	18 (56.3%)	8 (47.1%)	10 (66.7%)	0.448
LRYGB, n (%)	14 (43.8%)	9 (52.9%)	5 (33.3%)	0.448
Duration of surgery (min.), mean (SD)	117.66 (42.07)	112.94 (47.47)	123.00 (38.40)	0.519
Intraoperative complications, n (%)	0 (0.0%)	0 (0.0%)	0 (0.0%)	-
Postoperative complications, n (%)	4 (12.5%)	3 (17.6%)	1 (6.7%)	0.603
Reoperations, n (%)	2 (6.7%)	1 (5.9%)	1 (6.7%)	1.000
Rehospitalisations, n (%)	3 (9.4%)	1 (5.9%)	2 (13.3%)	0.589
LOS (days), median (Q1-Q3)	3 (2–4)	3 (2–4)	3 (3–4)	0.370

The median weight 6 months after the surgery was significantly lower in the group 1 – 84.00 vs. 105.00 kg (*p* = 0.005). Mean BMI after surgery also was significantly lower in group 1—32.17 vs. 38.64 kg/m2 (*p* < 0.001). TWL, EWL and EBMIL were higher in this group (*p* < 0.001) (Table 4). When it comes to the surgery effects lasting after 12 months, the differences between the groups were still significant with the same trend. BMI after 12 months was lower in group 1—32.44 vs. 37.32 (*p* = 0.012).

**Table 4:** Operation effects

Characteristic	All (*n* = 32)	Group 0 (*n* = 17)	Group 1(*n* = 15)	*p*-vaule
Weight 6 months after surgery (kg). median (Q1-Q3)	100.00 (84.38–118.00)	105.00 (98.50–137.50)	84.00 (80.00–104.00)	**0.005**
BMI 6 months after surgery (kg/m2). mean (SD)	35.61 (5.71)	38.64 (4.56)	32.17 (4.96)	** < 0.001**
TWL (%) after 6 months. mean (SD)	27.20 (7.61)	22.51 (5.54)	32.51 (6.02)	** < 0.001**
EWL (%) after 6 months. median (Q1-Q3)	49.41 (42.41–56.45)	44.64 (35.67–46.09)	57.18 (52.50–74.93)	** < 0.001**
EBMIL (%) after 6 months. median (Q1-Q3)	55.48 (48.65–64.79)	50.12 (39.67–53.32)	65.31 (60.44–88.75)	** < 0.001**
BMI 12 months after surgery (kg/m2). mean (SD)^a^	34.83 (5.40)	37.23 (4.68)	32.44 (5.12)	**0.012**
TWL (%) after 12 months. mean (SD)^a^	28.59 (7.79)	25.18 (7.98)	32.00 (6.10)	**0.014**
EWL (%) after 12 months. median (Q1-Q3)^a^	50.50 (45.19–59.28)	45.38 (38.18–47.92)	57.18 (50.51–74.93)	** < 0.001**
EBMIL (%) after 12 months. median (Q1-Q3)^a^	56.97 (50.54–67.57)	51.35 (46.06–57.05)	65.31 (56.89–88.75)	** < 0.001**

^a^Data available for 15 patients in Group 0 and 15 patients in Group 1 (2 patients lost to follow-up)

### Duodenal microbiota

**Fig. 2:**
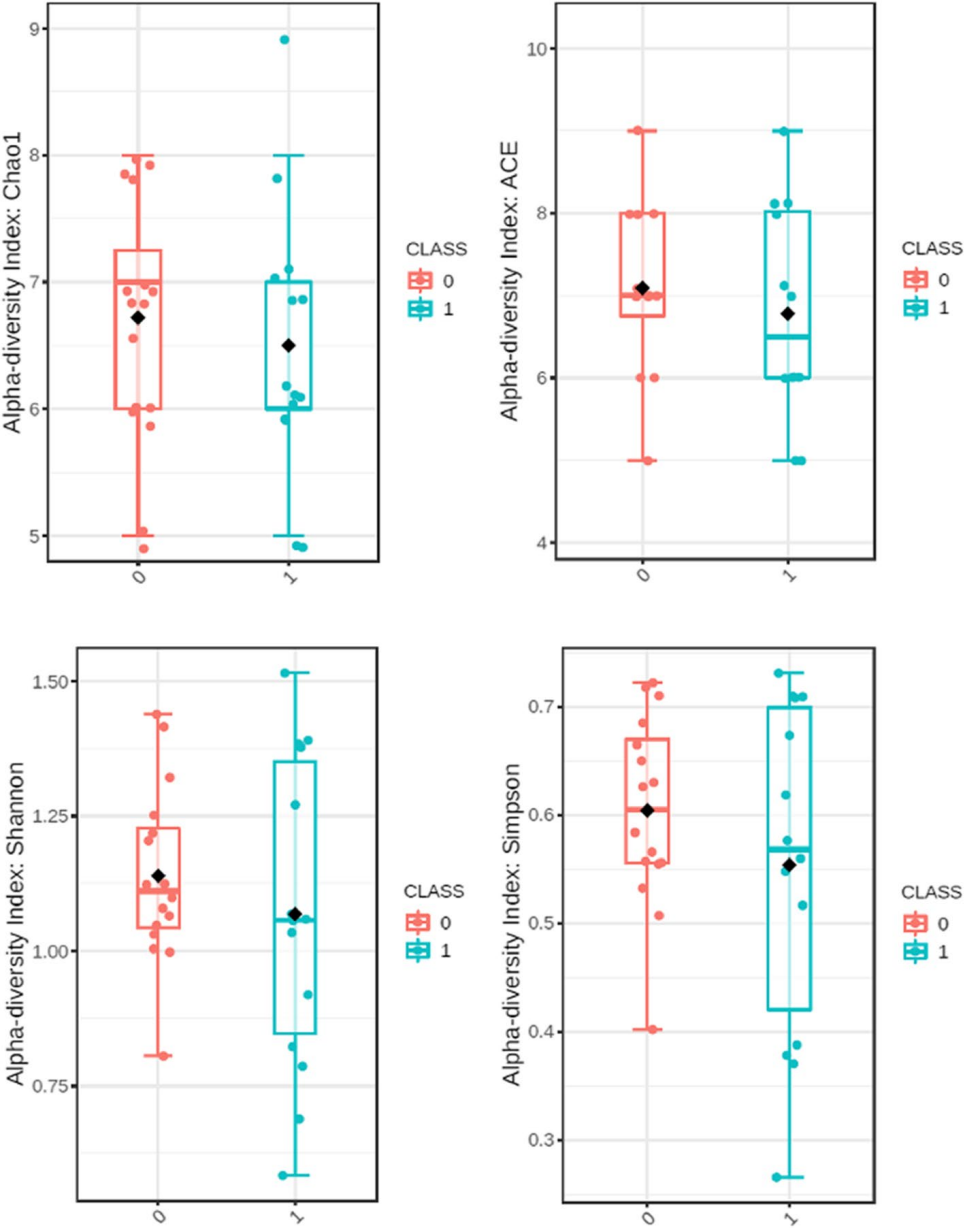
Alpha diveristy in phylum. Class 0 – group 0; class 1 – group 1

**Fig. 3:**
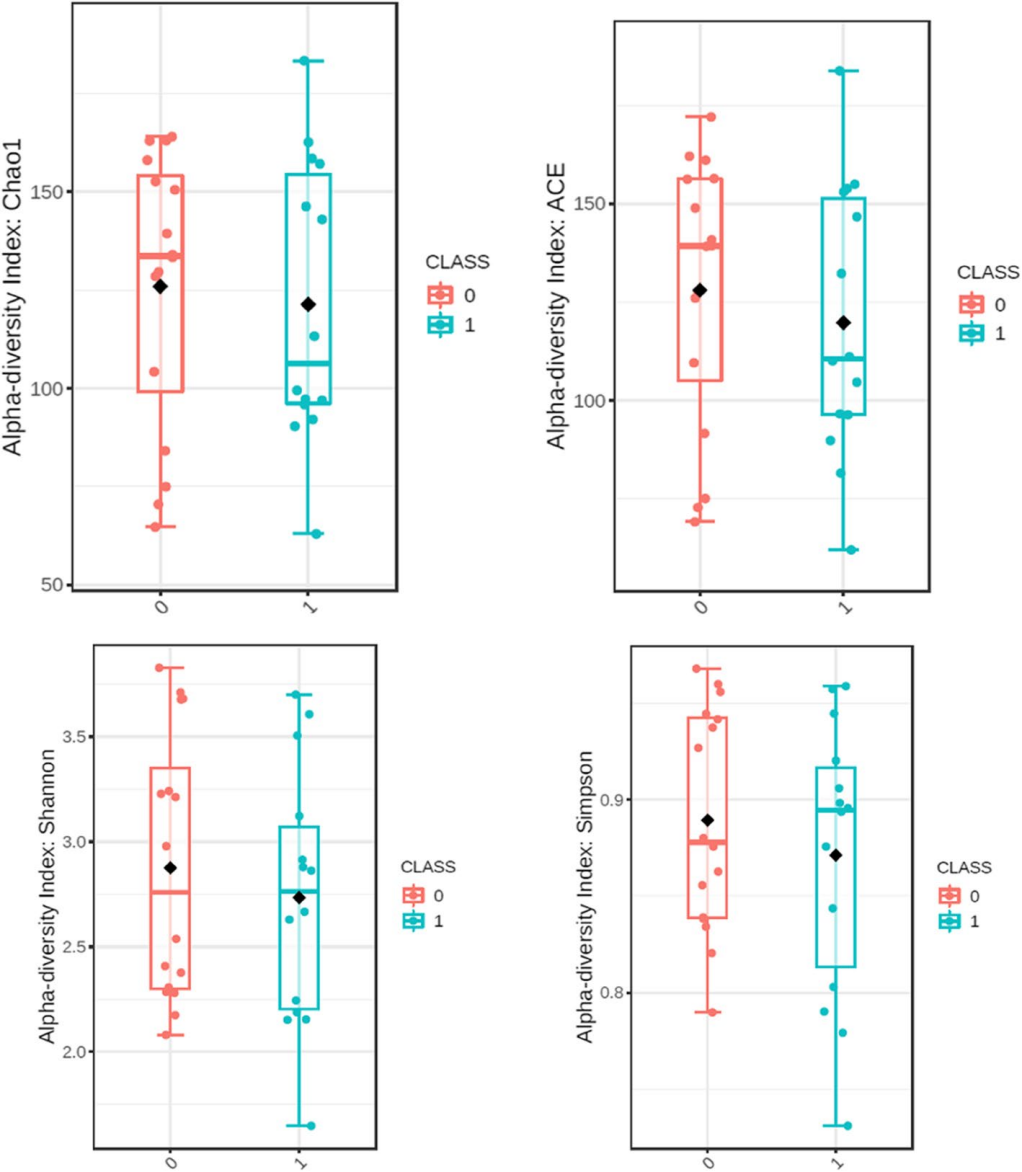
Alpha diversity in species. Class 0 – group 0; class 1 – group 1

**Fig. 4:**
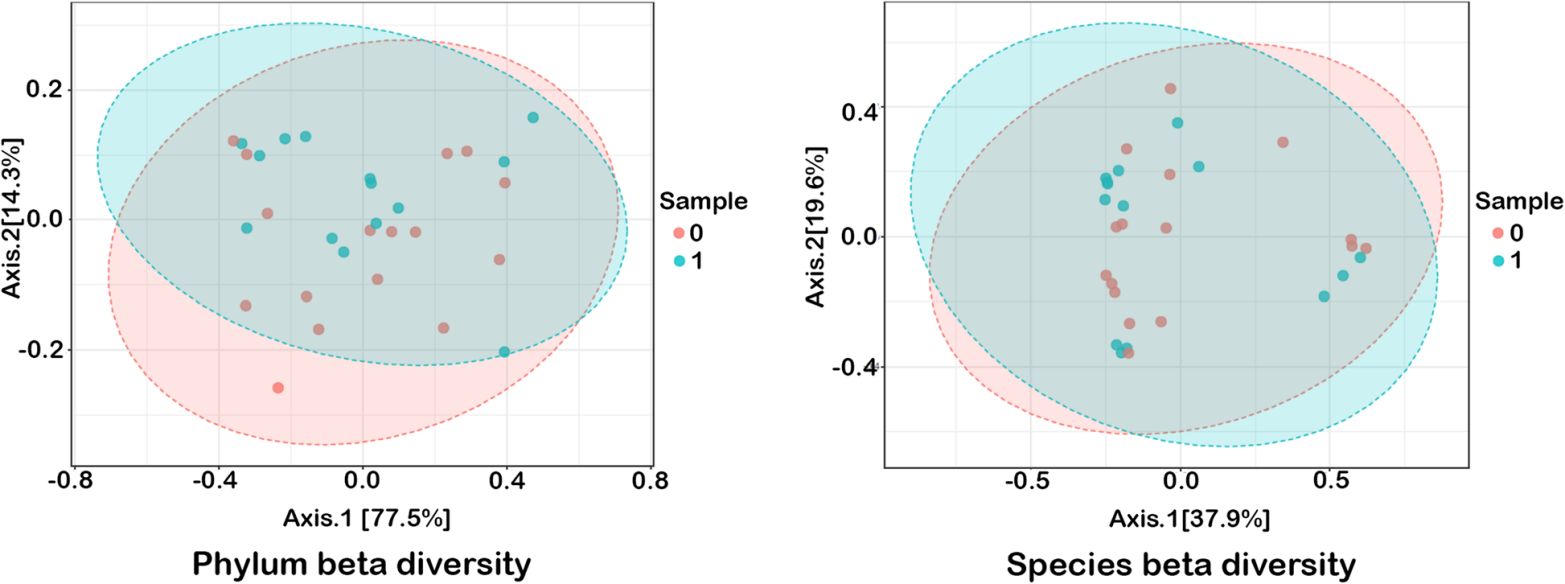
Beta diversity. Sample type 0 – group 0; sample type 1 – group 1

Alpha diversity presented as Chao1, ACE, Shannon and Simpson indices concerning phylum is presented in Fig. 2 and concerning species in Fig. 3. Beta diversity including Bray–Curtis index in both taxa is presented in Fig. 4.

**Fig. 5:**
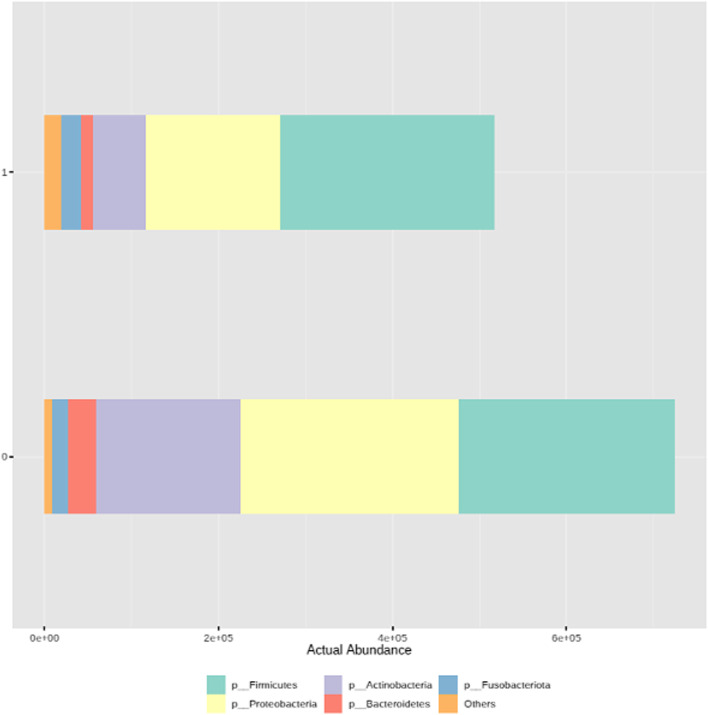
Median of total actual abundance

**Fig. 6:**
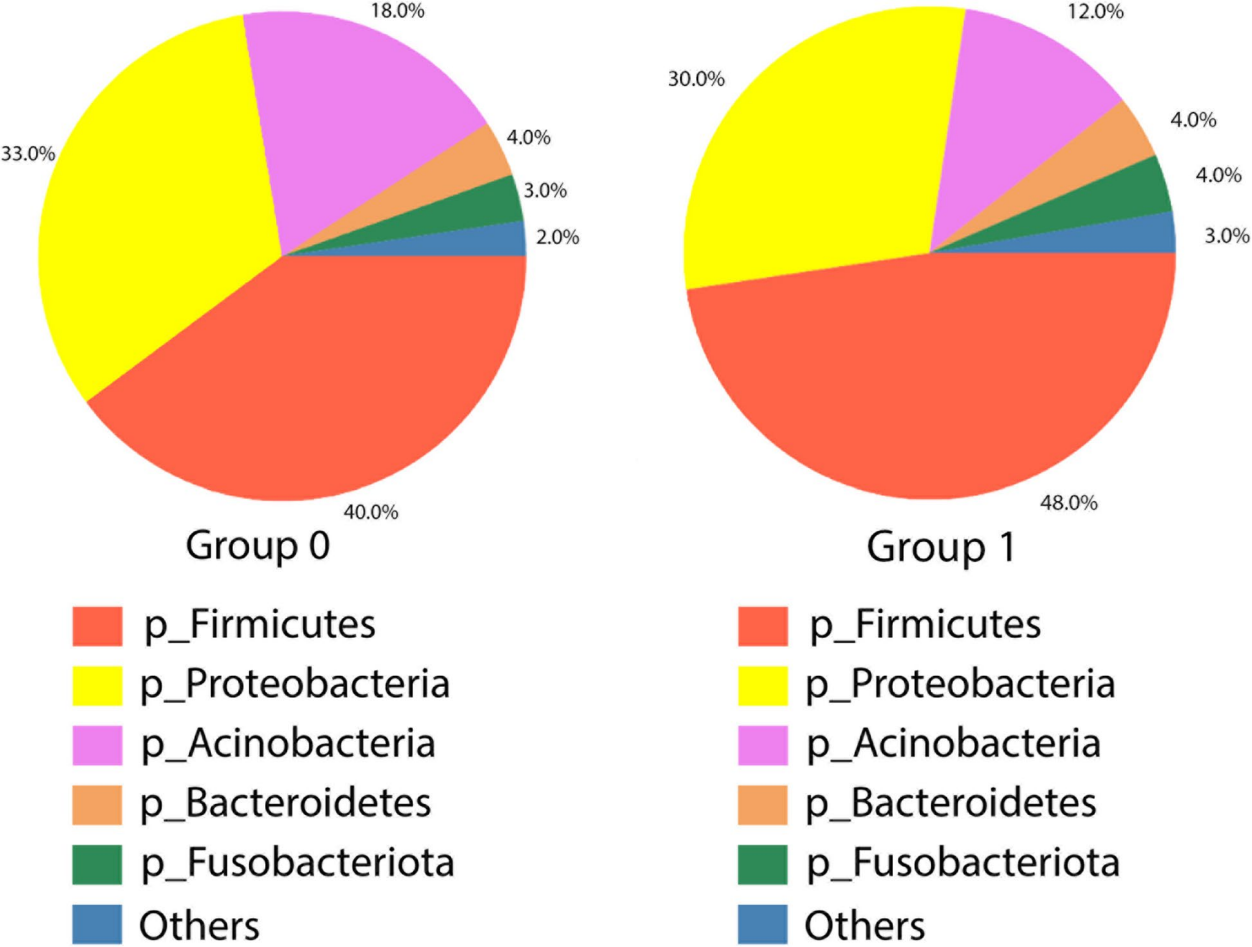
Contribution of bacterial phyla in duodenal microbiota of group 1 and group 0

There was a noticeable difference in the Median of Total Actual Abundance on phylum level (Fig. 5). Total Actual Abundance was higher in group 0. Specific phyla contribution was not significantly different between groups. The analysis of contribution showed 40% of Firmicutes, 33% of Proteobacteria, 18% of Actinobacteria, 4% of Bacteroidetes and 3% of Fusobacteriota in group 0 (Fig. 6). Whereas, in the group 1 composition of duodenal microbiota included 48% of Firmicutes, 30% of Proteobacteria, 12% of Actinobacteria, 4% of Fusobacteriota and 3% of Bacteroidetes (Fig. 6). There was a significantly higher amount of Roseburia and Arthrobacter in group 0 (*p* = 0.024, *p* = 0.027, respectively).

**Fig. 7:**
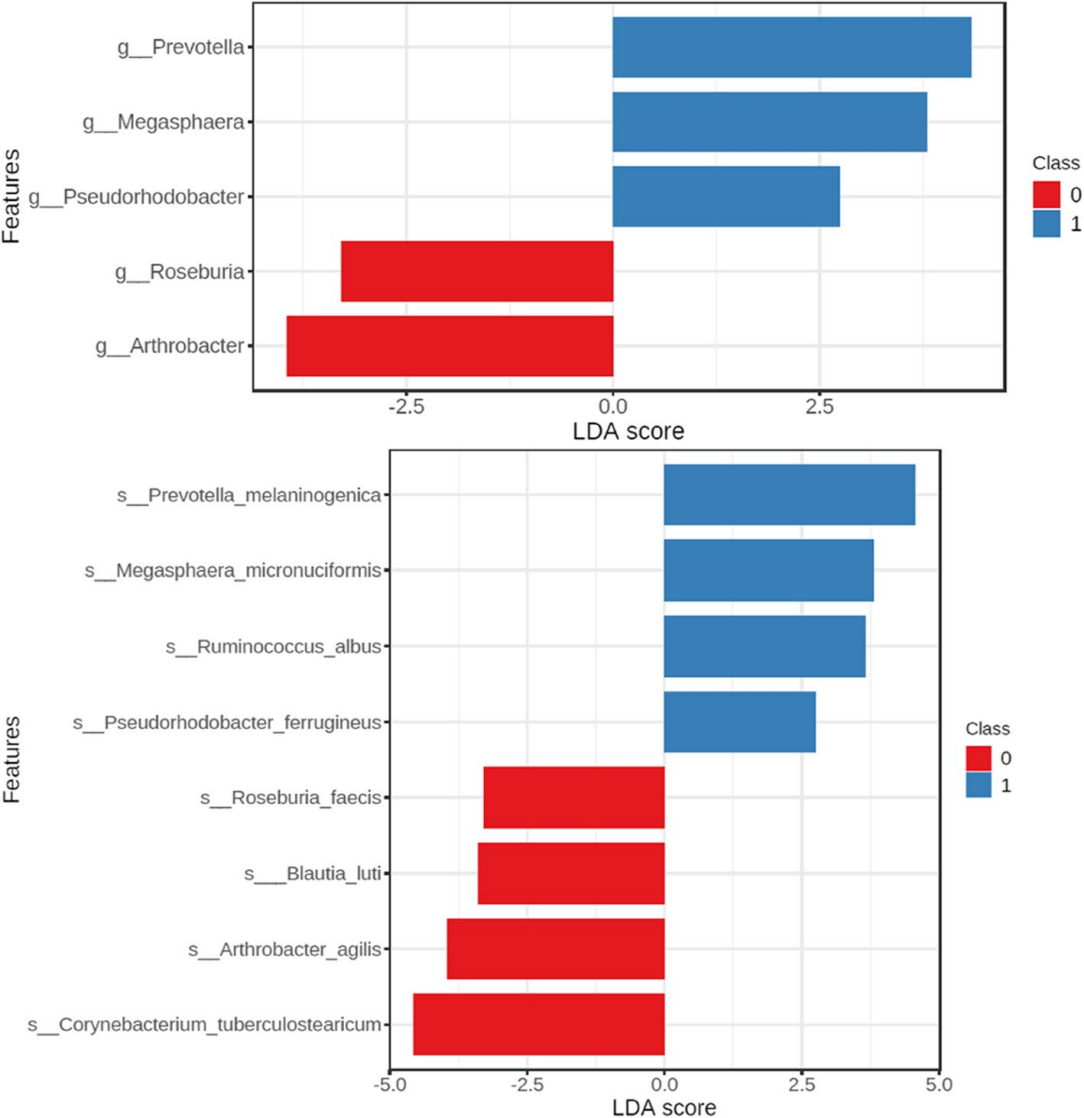
Linear Discriminant Analysis (LDA) effect size in genus and species. Class 0 – group 0; class 1 – group 1

LDA effect size analysis on genus level showed 5 genera to best explain the differences between the populations. Those genera are: Prevotella, Megasphaera and Pseudorhodobacter in group 1; Roseburia and Arthrobacter in group 0. Two species matching the previous genera was found to be more abundant in group 0 – Roseburia faecis and Arthrobacter agilis (*p* = 0.024; *p* = 0.027, respectively). Species LDA effect size analysis revealed 5 species from previously found genera and 3 species (Ruminococcus albus, Blautia luti, Corynebacterium tuberculostearicum) from other genera to be significantly more abundant (Fig. 7).

**Fig. 8:**
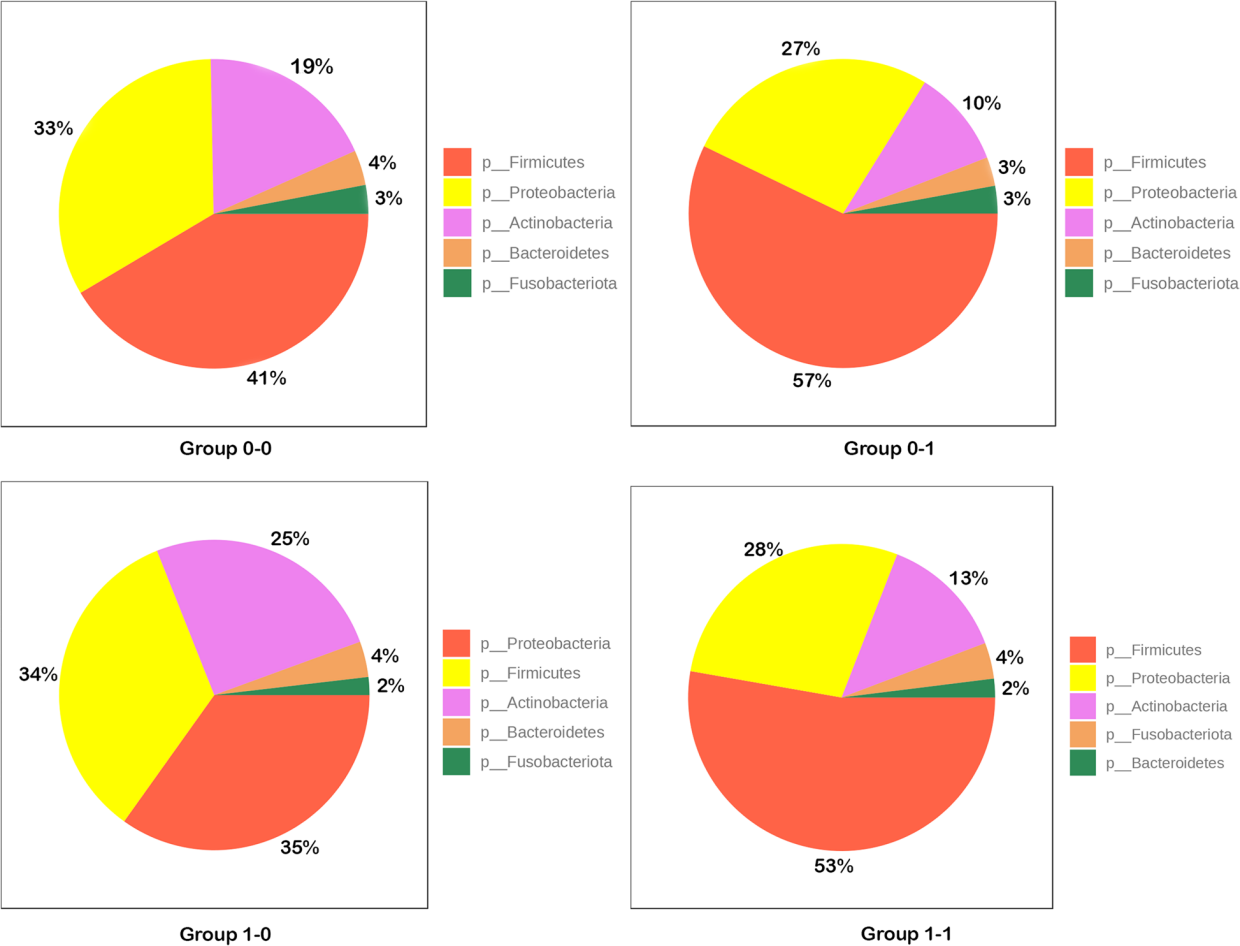
Contribution of bacterial phyla in duodenal microbiota in group 0–0 (group 0 after SG), group 0–1 (group 1 after SG), group 1–0 (group 0 after RYGB), group 1–1 (group 1 after RYGB)

**Fig. 9:**
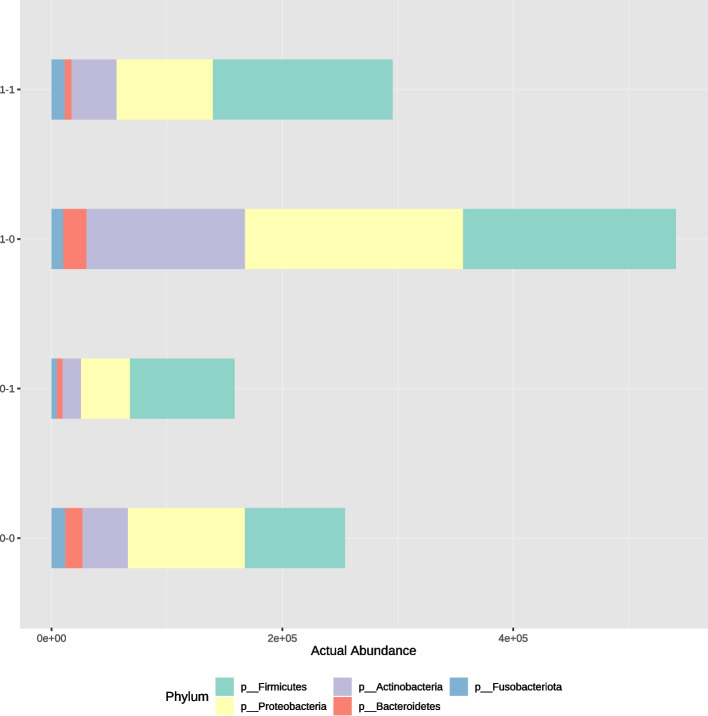
Median of total actual abundance in group 0–0 (group 0 after SG), group 0–1 (group 1 after SG), group 1–0 (group 0 after RYGB), group 1–1 (group 1 after RYGB)

**Fig. 10:**
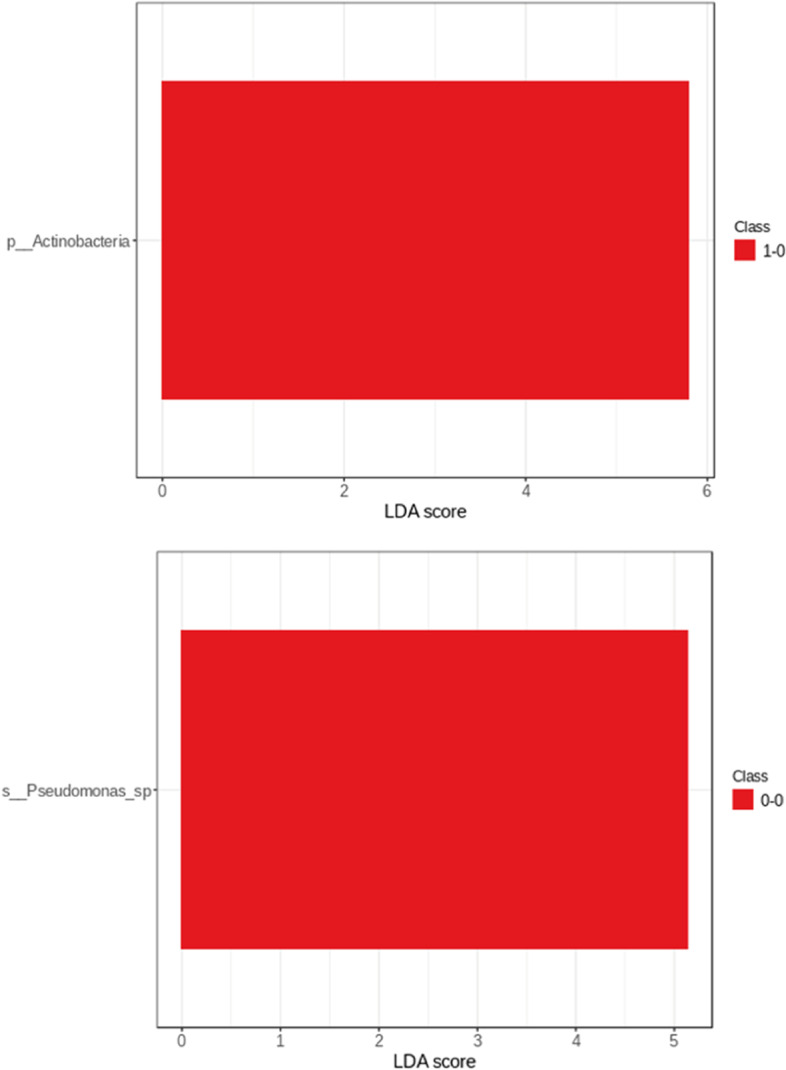
Linear Discriminant Analysis (LDA) effect size in phylum and species. Class 1–0 (group 0 after RYGB), class 0–0 (group 0 after SG)

Microbiota composition depending on different operation type is provided in Fig. 8. Among patients from group 0 after SG phylum Firmicutes dominated (41%), whereas after RYGB Proteobacteria dominated (35%). In group 1 after both SG and RYGB Firmicutes had the largest share in the composition (57% and 53%). Abundance of each phylum in analysed subgroups is presented in Fig. 9. On phylum level, LDA effect size analysis revealed that Acinetobacter was more abundant in the group 0 after RYGB than in other groups. On species level, Pseudomonas was significantly more abundant in group 0 after SG than in other groups (Fig. 10).

## Discussion

The following study is one of the first attempts to analyze microbiota composition of the duodenum in patients undergoing bariatric surgery, and how it varies between the successful and unsuccessful weight-loss groups following bariatric procedure. It should be noted that the following paper concerns a novel approach and still much research is needed. The role of our study is to pave the way for larger research conducted on grander scale to evaluate our findings and look for feasible practical applications. We believe that by following this route we can significantly further our understanding of factors determining the outcomes of bariatric surgery and the effects of treatment of obesity.

Several studies show that the microbiota of the human body has a great impact on the function of the “host organism”. Its’ composure, among many, has been linked to arthritis, colorectal cancer, development of type 2 diabetes [13, 14]. Moreover, factors such as lifestyle choices lead to alterations in the composure of the microbiota [15]. In the last decade, great efforts have been made to examine the relationship between gut microbiota and development of obesity. It has been determined that the ratio of Firmicutes to Bacteroidetes is one of positive predictors of development of obesity with the amount of Bacteroidetes varying between low (obese patients) and high (anorexic) [16]. The relationship between gut microbiota and prevalence of obesity is quite well understood, however there is much to be learned about the role it plays in determining the outcome of bariatric surgery.

Duodenum has been chosen for several reasons. Firstly, in the literature, microbiological analysis of upper parts tract, especially duodenum in the progress of obesity or diabetes, is rarely the subject of discussion [17]. This in turn is caused by the fact that biopsy collection is an invasive intervention that requires gastroscopic procedure [18]. Few studies that actually concern the analysis of duodenal microbiota mainly focus on paediatric patients with celiac disease [19–21]. Secondly, we have decided to focus on this section of the tract as the duodenum is located at the intersection between the stomach, secreting digestive enzymes, and the jejunum and ileum, which absorb nutrients. Its strategic position and role related to the digestive process and the absorption of nutrients made this section is worth investigating.

The only statistically significant difference in the group characteristics was mean age, which was lower for the successful group (group 1), this observation reflects the general consensus, that older age is a negative predictive factor for the favourable outcome of the bariatric surgery [22]. Other than the age difference the two groups are comparable with respect to demographic characteristics. Our perioperative data shows no differences between the groups, which could be expected, as the success/failure is measured as a long-term outcome.

When it comes to the composition of the duodenal microbiota, one of the significant results obtained in our analysis is the difference in prevalence of Roseburia and Arthrobacter which was higher in group 0 (*p* = 0.024, *p* = 0.027, respectively). Roseburia are members of the commensal microbiota of the intestine, they produce short chain fatty acids which influence colonic motility and reduce inflammation. Alterations to its prevalence have been previously linked to such diseases as type 2 diabetes, irritable bowel syndrome and obesity [23]. Arthrobacter has not been extensively researched as a marker, and there is little to no information on its role in obesity and as a prognostic factor in bariatrics. These observations were also supported by our further statistical analysis, including LDA effect size analysis. This analysis revealed that the group 1 is mostly more abundant in Prevotella, Megasphaera and Pseudorhodobacter genera. Increased abundance of Prevotella has been previously linked to Dietary-Fiber induced improvements in glucose metabolism [24], weight loss responses to specific diets and obesity management [25]. Megasphaera’s role the gut microbiota has not been thoroughly researched yet, some studies showed that its lowered abundance correlates with severity of Diarrheal Cryptosporidiosis [26] while others focus on its role in female reproductive health [27]. Literature search for the role of Pseudorhodobacter in gut microbiota and in the gut brain axis returned no studies.

Seekatz et al. conducted an analysis of duodenal microbiota in healthy individuals finding that it is dominated by *Firmicutes* which is in line with obese patients from our study. However, second phylum in the composition was *Bacteroidetes* while in our study *Bacteroidetes* accounted for only 3% of microbiota composition in both groups [28]. Moreover, another study showed that *Veillonella* sp., *Lactobacillus* sp. and *Clostridium* sp. are predominant in duodenum – all belonging to the *Firmicutes* [29].

We did not have access to the information about *H. pylori* presence in the patients and it was shown in the previous research that it might influence the microbiota composition with the increase of alpha and beta-diversity. On the other hand, the pattern of microbiota composition did not differ significantly in the infected patients [30]. Furthermore, eradication therapy may also influence the microbiota composition with the increase of *Proteobacteria*. Nevertheless, the relative abundance of all phyla restored to the baseline level after 8 weeks [31]. Thus, in our opinion *H. pylori* presence status in patients included in our study should not have influenced the results but it is a limitation of our study.

One of the most important limitations of our study arises from the fact that duodenal microbiota is a dynamic entity, which responds to any external stimuli such as changes in diet, alcohol intake, exercise and more. As described in the *Materials and Methods* section we gathered samples from each patient only once therefore our research is unable to account for both previous and future changes in the microbiota of patients, that also could have an impact on the success of the surgery [32]. This limitation arises from lack of sufficient funding that would allow us to repeat the sample collection at a different point in time. We did not gather the information about comorbidities after the surgery. Importantly, we should not treat microbiota as the only determining factor of the postoperative outcome, as it depends among other on individual skill of the surgeon, postoperative diet, present comorbidities, however all of them except for the mean age of patients were standardized and no difference was observed [33]. Therefore, to increase precision and validity of our observations, further research conducted on a larger population is needed. Also, there is a possibility that the type of bariatric procedure the patients underwent impacts the weight loss outcomes, and our research could be extended to include other operative techniques [34]. If sufficient funding was provided, statistically significant group for each of the procedures could have been gathered. However, as this was a pilot study, our goal was to establish whether any differences are present between the successful and unsuccessful groups. Moreover, there were more patients with joints problems in group 0. Although there was no significant difference between the prevalence of those problems between the groups, it might have affected the physical activity of the patients.

## Conclusions

The following study is one of the first attempts to analyze microbiota composition of the duodenum in patients undergoing bariatric surgery, and how it varies between the successful and unsuccessful weight-loss following bariatric procedure. Duodenal microbiota composition may be a prognostic factor for the success of the bariatric surgery but further research on the larger group is needed. LDA effect size analysis on genus level showed 5 genera to best explain the differences between the populations. Those genera are: Prevotella, Megasphaera and Pseudorhodobacter in group 1; Roseburia and Arthrobacter in group 0. Two species matching the previous genera was found to be more abundant in group 0 – Roseburia faecis and Arthrobacter agilis (*p* = 0.024; *p* = 0.027, respectively).

## Data Availability

The datasets generated and analysed during the current study are available in the portalwiedzy.cm-uj.krakow.pl/ repository, https://portalwiedzy.cm-uj.krakow.pl/info/researchdata/UJCM2487d902aba44a418407170a85fdfb37/Record%2Bdetails%2B%25E2%2580%2593%2BResearch%2Bdata%2B%25E2%2580%2593%2BJagiellonian%2BUniversity%2BMedical%2BCollege?r=researchdata&ps=20&tab=&lang=en.

## Abbreviations

WHO: World Health Organisation
SG: Sleeve gastrectomy
RYGB: Roux-en-Y gastric bypass
BMI: Body mass index
STROBE: Strengthening the Reporting of Observational studies in Epidemiology guidelines
ASA: American Society of Anaesthesiologists Classification
%EBMIL: Percentage excess BMI loss
%EWL: Percentage excess weight loss
%TWL: Percentage total weight loss
SD: Standard deviation
Q1-Q3: First and third quartile
LDA: Linear discriminant analysis
GERD: Gastroesophageal reflux disease
